# Illusionism Big and Small: Some Options for Explaining Consciousness

**DOI:** 10.1523/ENEURO.0210-24.2024

**Published:** 2024-10-25

**Authors:** Michael S. A. Graziano

**Affiliations:** Department of Psychology and Department of Neuroscience, Princeton University, Princeton, New Jersey 08544

**Keywords:** attention, consciousness, global workspace, illusionism, integrated Information, visual awareness

## Abstract

Illusionism is a general philosophical framework in which specific theories of consciousness can be constructed without having to invoke a magical mind essence. The advantages of illusionism are not widely recognized, perhaps because scholars tend to think only of the most extreme forms and miss the range of possibilities. The brain's internal models are never fully accurate, nothing is exactly as the brain represents it, and therefore some element of illusionism is almost certainly necessary for any working theory of consciousness or of any other property that is accessed through introspection. Here I describe the illusionist framework and propose six specific theories. One purpose of this article is to demonstrate the range of possibilities in a domain that is not yet sufficiently explored. The second purpose is to argue that even existing, popular theories, such as the integrated information theory or the global workspace theory, can be transformed and greatly strengthened by adding an illusionist layer. The third purpose is to argue that when illusionist logic is used, even very disparate theories of consciousness that emerge from unrelated conceptual origins begin to converge onto a deeper, unified understanding.

## Significance Statement

Illusionism is a philosophical framework in which consciousness is not exactly what we think it is. The brain's internal models are never perfectly accurate, and therefore nothing is exactly as the brain represents it. The conceptual advantages of illusionism are not widely recognized, perhaps because scholars tend to think only of the most extreme forms and miss the range of possibilities. This article describes the illusionist framework and proposes six specific theories within it. One purpose is to demonstrate the range of possibilities in a domain that is not yet sufficiently explored. Another purpose is to show that with illusionism, even very different theories begin to converge toward a deeper, unified understanding of consciousness.

*For Dan Dennett, who put illusionism on the map.*

## Introduction

The term “illusionism” gives the impression of a theory that dismisses the entire phenomenon of consciousness. Indeed, some forms of illusionism do exactly that, while other forms are more cautious (Gazzaniga, 1970; Nisbett and Wilson, 1977; Dennett, 1991; Blackmore, 2003; Frankish, 2016). I argue that illusionism should be seen as an umbrella term. It is not a specific theory of consciousness, so much as a general philosophical framework in which many theories can potentially be constructed. To my knowledge, however, the only cognitive neuroscience theory in the literature that is overtly illusionist is the Attention Schema Theory (AST), proposed in 2011 (Graziano and Kastner, 2011; Graziano, 2013; Webb and Graziano, 2015; Graziano et al., 2020). It would be beneficial to build other illusionist theories to serve as healthy competition to AST and to show the range and power of the general approach. Moreover, many already existing theories of consciousness can be seamlessly retrofitted with an illusionist layer, potentially greatly strengthening them. The powerful conceptual advantages of illusionism are not widely recognized, perhaps because scholars tend to think only of the most extreme form and miss the range of possibilities.

Broadly speaking, in illusionism, consciousness is not exactly what we think it is (Gazzaniga, 1970; Nisbett and Wilson, 1977; Dennett, 1991; Blackmore, 2003; Frankish, 2016). The brain's internal models are never perfectly accurate, and therefore absolutely nothing is exactly as the brain represents it. In the illusionist perspective, the reason why explanations of consciousness have been elusive is that scholars are mistakenly attempting to explain the subtleties of something that does not have the form they think it does.

The modern study of consciousness tends to focus on how the brain generates an intangible essence of experience. Yet the quality of experience was not always the target of consciousness research. More than a hundred years ago, James (1890) coined the term “stream of consciousness.” To him, consciousness was a stream of content—thoughts, decisions, sensory impressions, and memories. In principle, anything that had that stream of content was conscious. Then, in the middle twentieth century, computers began to encroach on the domain of consciousness. In 1950, for example, Turing (1950) wrote his famous paper on whether computers can think. Bit by bit, computers began to take on many of the components that are supposed to make up the stream of consciousness. They could process sensory input, store and retrieve memory, make decisions, and can now do even more. I suggest that this gradual encroachment of technology and computation over the decades threatened people and led to a rising antitechnology discomfort. That discomfort may have motivated scholars to define something special about the conscious mind that can be dissociated from mere computable content. Nagel (1974) wrote about experience, the “what-it-is-like” part, and Chalmers (1995) wrote about the “hard problem” as distinct from the “easy problem.” When you look at an apple, you do not just process the shape and color like a computer. You have a special, intangible essence, an internal experience. By now, almost all theories of consciousness accept, as a fundamental assumption, the existence of the what-it-feels-like essence. (For an extensive survey of existing theories, see Blackmore and Troscianko, 2018; Doerig et al., 2021) In that framework, the task of a theory is to explain how phenomenal experience is generated, or at least to specify the conditions under which it exists.

In illusionism, however, the question of consciousness is different. Imagine that a house is photographed and the picture is run through too many photocopies, resulting in a black-and-white, blurry, almost unrecognizable representation. To understand that picture, our first job is to avoid interpreting it too literally and mistakenly assuming that there exists an actual, 3-inch-tall, two-dimensional house with smudgy boundaries. We must be sleuths and identify the original building that is the subject of that representation, and we must understand the steps by which that building was rendered into a distorted representation. Just so, imagine an unknown something—whether substance or process—that is rendered into a representation by the brain's imprecise information embeddings, then run through higher semantic manipulation, and then run through speech production. It comes out the other end of this chain of depictions and copies of depictions as the claim that we have an ethereal magic of consciousness inside of us. As scientists, as consciousness researchers, can we figure out what the original item is? Can we figure out its specific adaptive uses in the brain and the steps by which it became represented in an imprecise or schematic manner? And just how different is the real item from the consciousness that we claim to have? In this context, it has been suggested that the term “caricature” might be a more apt descriptor of consciousness than “illusion” (Graziano, 2019). To most people who first encounter the phrase, “consciousness is an illusion,” the term seems to imply that there is nothing present and the brain has made a mistake—in effect, the picture of the house is a fake and no house exists. This is probably not exactly what most scholars mean when they use the term illusionism, and yet it tends to be the message that readers take. Caricature may be a better term, because it more clearly implies that there is, actually, a real subject of the representation, that our understanding of that item is distorted rather than entirely wrong, and that the whole phenomenon is not just a brain mistake but serves a useful purpose.

In this article, I will first describe a framework for thinking about the problem of consciousness from a representation point of view, emphasizing information flow through neural systems. I will then propose and discuss six different hypotheses about “Item 1,” the unknown thing from which human claims about consciousness stem. Each of these hypotheses represents a different illusionist theory. Some are extreme forms that are unlikely to be correct, but I present them to show the range of possibilities. Some are closely related to common theories in the current literature. For example, Hypothesis 3 is an illusionist version of the integrated information theory (IIT; Tononi, 2008; Tononi et al., 2016). Pairing IIT with illusionism may seem like odd bedfellows to those who know, and yet the hypothesis makes some interesting rational sense, showing just how versatile and useful illusionism can be. Likewise, Hypothesis 4 is an illusionist version of the global workspace theory (GW; Baars, 1988; Dehaene, 2014; Mashour et al., 2020). Hypothesis 5 is AST (Graziano and Kastner, 2011; Graziano, 2013; Webb and Graziano, 2015; Graziano et al., 2020).

The most remarkable feature of these illusionist theories may be that so many of them converge. Despite their different origins, Hypotheses 3, 4, and 5 are less like alternatives and more like keyhole perspectives on one underlying theory. In the final section of the paper, I describe how the illusionist approach may open a window on a deeper, unified framework for consciousness.

## From Vision to Speech

In this section, I analyze a particular instance of verbal behavior. The goal is to lay out a story that is uncontroversial to neuroscientists, at least in its general outlines. I will then shift to the topic of consciousness and ask how far one can proceed using the same concepts.

**Figure 1.**
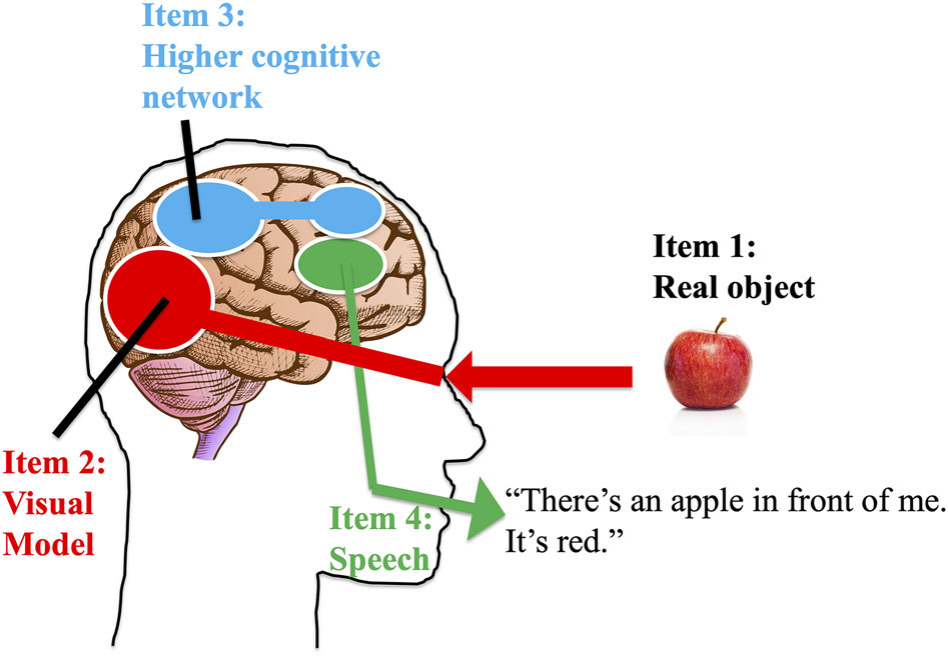
Highly schematized account of the transformation from visual input to verbal output. Light reflecting from an apple (Item 1) is transduced into neural signals. The neural information is rendered into an embedding or a model (Item 2) which consists of a complex pattern of activity among neurons. The visual model influences a higher cognitive network (Item 3) that incorporates semantic embeddings. The higher cognitive network influences a speech network (Item 4) that constructs the correct neuronal output signals to activate speech muscles and produce Bob's verbal utterance.

In Figure 1, Bob tells us, “There's an apple in front of me. It's red.” This speech output is labeled Item 4. One can work backward from Item 4 to provide an overarching explanation for the behavior. Speech muscles are controlled by spiking neuronal activity mainly in the hypoglossal nerve, though other nerves contribute. Those nerve outputs are under the control of a broad speech network including the facial nucleus, motor cortex, Broca's area, Wernicke's area, and so on (Hagoort, 2017). The details are not important to the present argument, and Figure 1 schematically highlights only one area. What is important here is that neurons in a speech-related network are active in a complex pattern, causing Bob's speech output.

The thought behind the utterance is probably not constructed in speech-specific areas, but instead in deeper layers that might be loosely called higher cognitive areas. These areas are shown schematically in Figure 1 as a parietofrontal network, though presumably the cognitive network is larger and richer. It is labeled here as Item 3. In the modern vocabulary of machine learning, one might say that a set of semantic concepts relating to apples and color have embeddings in this cognitive network. An embedding is a pattern of activity among neurons, a code that in this case stands for semantic concepts. That pattern influences the pattern of activity in the speech network, which in turn drives a pattern of muscle activity, which makes vocal sounds come out of the mouth.

Yet Bob's realization about the apple is not just a semantic concept originating in his higher cognition. Instead, his higher cognitive network is informed by his visual system, labeled here as Item 2. The pattern of activity among its neurons represents visual events, in this case a red apple in a location in front of Bob. Here I will also call that visual embedding a model in keeping with previous terminology. The visual system has constructed a bundle of information, a model, that says in effect, not in words of course but in neurally encoded information, “There's an apple, it's round, it's red, it has a dent here, it has a brown spot there,” and so on.

Ultimately, the stimulus information in the visual system derives from an object in the environment, labeled here as Item 1. Light reflects off the apple, enters the eyes, causes neuronal activity in rods and cones, and initiates a cascade of information into the visual network. It is worth making a point that may seem trivial but will return later. There must be an interface, a set of detectors, to take light from the environment and turn it into an information code; otherwise the brain would not know about the visual stimulus. Bob cannot form semantic concepts about it or talk about it, if it has not been turned into an information embedding. A second point worth making is that Item 2, the visual model, is low level in the sense that it is automatic. Higher cognition receives the visual information but has relatively little internal control over it. Bob cannot choose to make his visual system model a banana when he's looking at an apple. Of course, delusions and hallucinations are possible, but most of the time the model provides a constrained picture of reality. A third point worth making is that the visual model is not fully accurate. It is necessarily a simplification. A neural embedding is an efficient, reduced way of representing something. For example, the apple does not really have a color. It has a complicated spectrum of reflected light. The eyes and brain compress that property of the world into a low-dimensional space that we call color. All models in the brain are schematizations and simplifications of the real items that they represent.

Figure 1 therefore depicts a neuroscientist's narrative, from visual input to motor output. There should be nothing controversial about this account. Even if some of the brain details turn out to be wrong as neuroscience progresses, and even though the reality is more complex and overlapped than depicted here, the general outline is almost certainly correct. There are inputs, information is encoded in progressively deeper layers, and there are outputs, resulting in Bob's specific claim. Equipped with this framework, I will now approach the topic of consciousness.

## From Self Model to Speech

**Figure 2.**
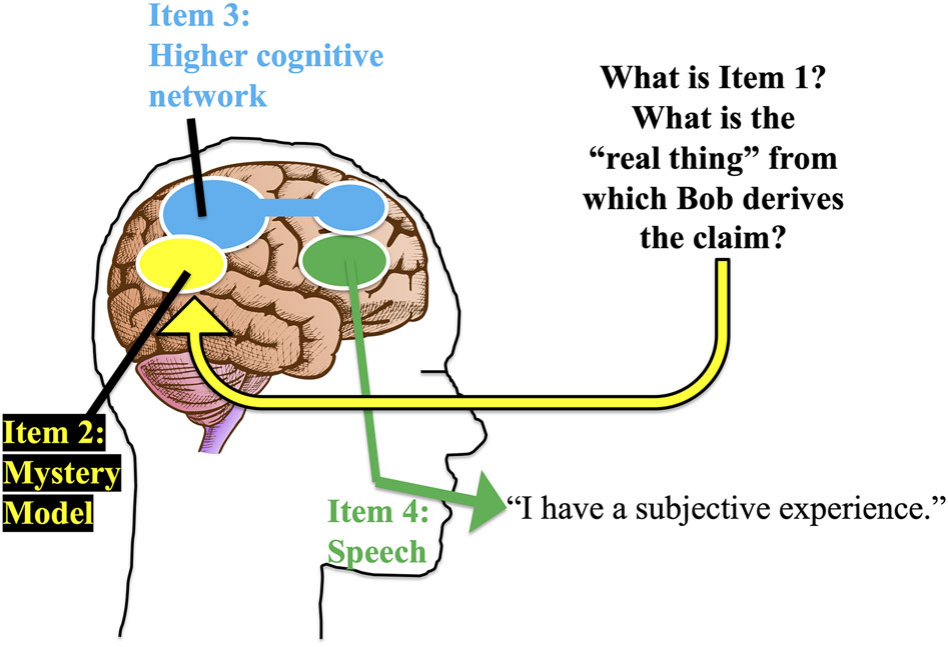
Highly schematized account of the transformation from an unknown Item 1 to the verbal claim that conscious experience is present. In Hypothesis 1, Item 1 is a magical mind essence. In Hypothesis 2, Item 1 does not exist. In Hypothesis 3, Item 1 is the integration of information. In Hypothesis 4, Item 1 is Item 3, in a recursive loop. In Hypothesis 5, Item 1 is attentional enhancement. In Hypothesis 6, Item 1 is the deep, selective processing of information in the brain, which is associated with integrated information, the global workspace, and attention.

In Figure 2, Bob makes a different statement. He says, “Not only is there an apple, but I have a subjective experience of it. There is something it feels like when I see the round shape and the red color. I have phenomenal, conscious experience.” Here I will follow the same conceptual framework as in Figure 1, working backward item by item, to outline how Bob, and philosophers, and me, and the readers of this article, can make that kind of claim.

Bob's speech output, Item 4, is caused by patterns of neural activity in his speech network. The activity in the speech network is in turn driven by patterns of activity in his higher cognitive network, Item 3. Yet Bob's realization about subjective experience is not just a passing semantic concept generated by higher cognition. Instead, the higher cognitive network must be informed by a more automatic, lower-level model, an Item 2, that encodes the presence of subjective experience.

One might ask, why do we need a model, a bundle of information that “tells” Bob that he is having a conscious experience? Why can't he just have the conscious experience? The reason is that it is not possible for Bob to construct semantic content, or to say it out loud, if his neural networks do not encode the relevant information. In analogy, if Bob believes there is an apple in front of him, and says there is, then he must have neurally encoded information in his brain that represents that apple. Just so, if Bob believes he has conscious experience, if he says he has it, then whatever conscious experience is or is not, Bob must have a neural embedding that represents it.

We do not know the details of this hidden model that tells Bob about the presence of consciousness in him. In Figure 2, I have centered it on the temporoparietal junction for reasons that emerged from my lab's empirical work (Kelly et al., 2014; Webb et al., 2016a; Wilterson et al., 2021), but the proposed location is not important to the present argument and it could be somewhere else or distributed across many areas. Whatever system constructs it, the information in the model depicts Bob's mental state of experienceness and that information becomes available to the higher cognitive network and from there to the speech network.

To be clear, the account so far is not a theory of consciousness. It is, instead, a framework for thinking about how people semantically know they have consciousness and how they can verbally claim to have it. It is a recognition that because we can talk about consciousness and because we have semantic content about consciousness, the brain must have an information embedding that depicts consciousness. We have yet to address the question: what is the event or object that informs the model, that informs higher cognition, that leads Bob to say he has a subjective, phenomenal experience? What is Item 1? Below I offer six hypotheses.

## Hypothesis 1: The Magical Mind and the Problem of Arrow B

In a traditional account, Bob has a “feeling” or experience associated with visually processing the apple—he has phenomenology. There are many proposed theories of how this phenomenal essence is generated by the brain (for review, see Blackmore and Troscianko, 2018; Doerig et al., 2021). All of them face a challenge that must be overcome.

**Figure 3.**
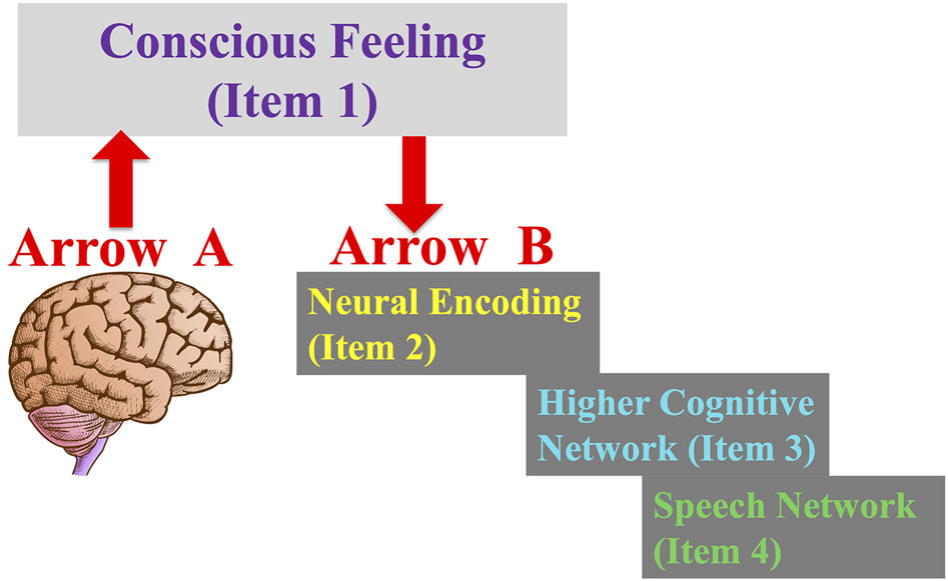
Arrow A and Arrow B. Arrow A is the first explanatory gap. How can physical neurons give rise to an ethereal feeling? Arrow B is the second, equally important, explanatory gap. Once the feeling is generated, how does it then physically impact neuronal activity, such that it can be transduced into a neural embedding or a model, such that higher cognition can form the semantic idea that it is present, such that people can say that they have it?

Figure 3 illustrates the challenge. Theories of consciousness almost always focus on how the activity of neurons can produce a conscious feeling, a problem sometimes called the explanatory gap or the hard problem (Levine, 1983; Chalmers, 1995). I call it Arrow A. There is, however, a second, rarely considered explanatory gap. How does the feeling, once it is produced, cause a specific information embedding in Bob's neural networks? I call this second process Arrow B (Graziano, 2013). The concept of Arrow B is in many ways similar to the “hard question” posed by Dennett, “And then what happens?” (Dennett, 2018). The feeling itself, the intangible, what-it-feels-like essence that emerges, would need to physically impact the activity of neurons, revving up this neuron and inhibiting that one, in such a specific pattern, that it imprints exactly the right information to represent itself. Otherwise, Bob's brain would not be able to contain the semantic idea that he has it, or construct the words to report that he has it. To implement Arrow B, neurons would need to have a type of receptor mechanism, never before discovered, that responds to the presence of subjective conscious feeling. To be clear, the challenge is not for these hypothetical neurons to respond to electric fields, or to oscillations, or to quantum states, or to the extent of integration of information, or to whatever physical mechanism one hypothesizes to ultimately cause the conscious feeling. Instead, these hypothetical neurons would need to respond to the emergent feeling itself—the what-it-is-like part. Like the rods and cones in the retina that turn light into neural activity, here you would need neurons that turn conscious feeling into a pattern of neural signals. These consciousness Geiger counters embedded in the brain begin to sound uncomfortably like pseudoscience.

**Figure 4.**
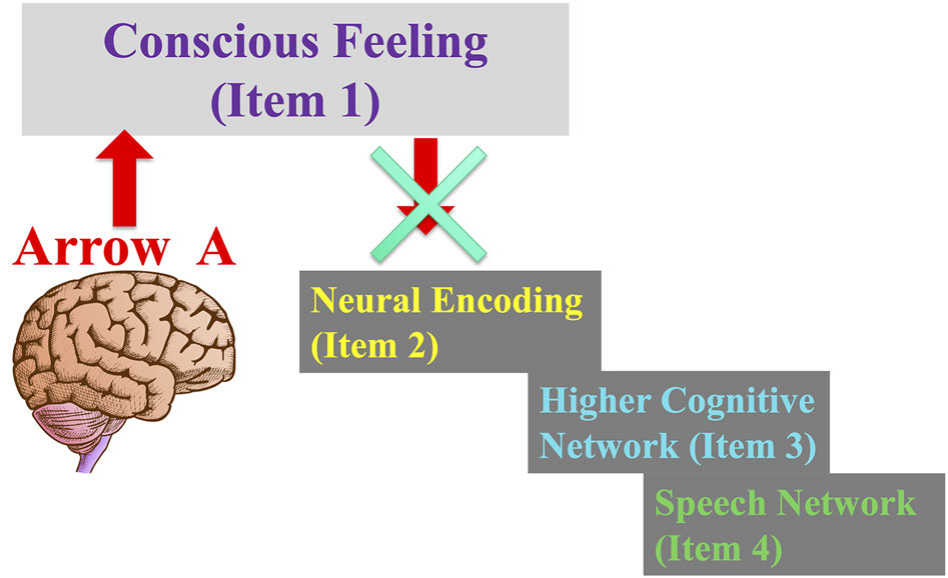
The causality is broken without Arrow B. If the brain has no mechanism for detecting conscious feeling, such that it can no longer be represented in a neural code, then people have no way to think about or talk about that consciousness. Without Arrow B, when people form the semantic idea that they have conscious experience, when they say they have conscious experience, and when they describe specific properties and features of conscious feeling, none of those events are based on an actual conscious feeling generated by the brain. Theories that propose an Arrow A but ignore Arrow B are not explanatory.

As shown in Figure 4, without the neurons that have consciousness receptors, without Arrow B, the causal link is broken and the conscious feeling that emerges from the brain cannot be the consciousness that we say we have. Without Arrow B, even if Bob's brain generates a magnificent conscious experience through Arrow A, it would not be the reason why Bob constructs the semantic proposition that he has consciousness, why he is so certain that he has consciousness, or why he says he has consciousness. Even the way he describes consciousness—the specific properties he says that it has—cannot be motivated by the actual properties of the consciousness in his head, as long as that causal link is broken.

The problem of the magical mind theory is therefore not so much Arrow A as it is Arrow B—the second explanatory gap. If a theory does not explain Arrow B—how neurons physically detect consciousness and thereby turn it into an informational embedding—then you do not have a working theory because it does not explain the known behavior.

It is powerfully tempting to say, “Bob believes he has consciousness, and claims to have consciousness, because he actually *does* have it.” Who can argue with that? But spend half a minute thinking about it, and you should realize that it does not work. Consider an analogy. Your brain has a complex pattern of blood flow, but as far as is currently known, does not translate that pattern into an information embedding. Or at least, if it does so, that model is not accessible to higher cognition. You cannot introspect and say, “My brain has a really nice blood pattern right now.” You do not have that automatic, internal model, moment-by-moment informing your higher cognition. Because blood flow is a physical process that could, in principle, be detected and transduced into a pattern of neural activity, this type of introspective insight could in principle have evolved. It appears not to have, since we don't have it. The lesson of the blood flow example is that, just because your brain has something in it, does not mean you know it, believe it, think it, or claim it. You can do those things only if the item in question is physically translated into a neuronal embedding, such that specific information is encoded in a specific way in the right neural networks. It does not work to say, “Bob believes and claims he has conscious experience because he does have it.” That argument is neither meaningful nor valid. It is magical thinking. It treats Bob's claim that he has consciousness as if it follows different rules from every other claim that Bob makes.

Could the magical mind theories be correct anyway? Suppose, for the moment, that Figure 3 is an accurate outline. By way of Arrow A, neurons produce a conscious feeling, a subjective experience, as almost all accounts assume. Suppose that some as-yet unknown mechanism for Arrow B exists. Conscious feeling itself somehow impacts neural networks, creating a specific information code, or model, informing Bob's cognitive machinery and his speech network, such that he can say that a conscious feeling is present. Assume for the moment that this account is correct. Even so, the feeling that Bob “knows” he has and says he has is entirely dependent on the information embedding—on Item 2. Whatever the model describes, that is what Bob is certain he has. There is no reason to suppose that the model is accurate. In fact, the opposite—it appears that all models in the brain are schematic, simplified, and inaccurate. Therefore, even if we accept the magical mind hypothesis, it is still the case that the consciousness arising from the brain is different from the kind of consciousness that Bob describes having. The conscious experience that you “know” you have, that philosophers are certain they have, that people tell each other they have and write books about having, is a phenomenon as it is described by a sketchy informational embedding. The nature of the real item, the real consciousness, remains unknown. The only description we have of it comes from an indirect route—through an imprecise model that informs a higher cognitive network.

Therefore, even if we try to make the traditional account work, the real consciousness that Bob actually has could just as well be something unexpected, different in its intrinsic properties from what either Bob or we believe it is—as different as, for example, the real city of New York is from a paper map of the city. Even the traditional account, when held to a basic standard of logic, turns into an illusionist account.

It is an uncomfortable insight, but the fact that Bob and you and I all “know” that we are having a phenomenological conscious experience right now is not evidence that we have it. Instead, it is strong evidence that our brains have an information embedding, a model, that depicts something about ourselves. The depiction may be so schematic and distorted that we are left not understanding the item that is being modeled.

If the magical mind theories are so irrational and riddled with not one but two fatal gaps, then why are they so popular? There is a reason why the magical mind theories are universally compelling and capture the majority of the field of study. They are verbalizations of, or at least they are consistent with, our automatic internal models. They resonate with our intuitions. People's convictions fit easily into that way of thinking and rebel at the alternative. There may also be a defensive component: magical mind theories help to protect the mystery of the human spirit against materialism and maybe especially against the uneasy growth of technology. Artificial deep neural networks in particular, which are capable of such spectacular successes by imitating aspects of the brain, turn out to be linear algebra machines, and it violates our natural intuitions—our self models—to think that the human brain is a collection of biologically-instantiated algebra machines. Scholars are therefore powerfully, ideologically motivated to search for magical theories of consciousness. I will go so far as to say that, in my view, the field of consciousness studies has become a Trojan horse for the entry of overt spiritualism into the discussions, the conferences, the journals, and the funding opportunities of professional science.

## Hypothesis 2: Hard Illusionism

Here I will describe the most extreme form of illusionism. Though I think it is unlikely to be correct, it helps define the conceptual boundaries.

The brain builds models of real things. The visual system builds models of objects in the world. The auditory system builds models of sounds. The body schema is a model of the physical body. These bundles of information represent things in the world that are useful to monitor and predict. But suppose that the brain anomalously builds a model that depicts Bob as having a conscious experience, and causes Bob's higher cognitive machinery to contain the semantic information that he has a conscious experience, and causes Bob to say that he has a conscious experience—but the model does not actually correspond to anything real? What if there is no Item 1? What if Bob's consciousness is entirely a fiction, depicted by an errant model? Maybe Bob learned this false model through cultural upbringing. Everyone else thinks they have consciousness, so Bob does too. In that case, consciousness is not just a false construct, but also limited to humans.

This hard illusionist account is not inconsistent with Bob's behavior, and therefore it should be considered as a possibility. However, for my part, I find it difficult to see how such a model would have emerged. Evolution shapes the brain to construct models that can monitor and predict real items in the world. It seems unlikely that evolution would have shaped the brain to waste its energy building models of things that do not actually exist. As far as is known, a similar model is present in almost all human brains across cultures. If it is an anomalous model of nothing at all, then the sheer scale and consistency of the bamboozlement seems improbable.

A less extreme form of illusionism might be more likely. Suppose there is an Item 1—there is a real thing that is being modeled—but the model is schematic and imperfect. Therefore, the consciousness that Bob describes having, the phenomenology or the intangible experience that he believes he has is somewhat different from the actuality of Item 1. This distinction between a model in the brain and the item that it models is necessarily true, as all neuroscientists should know. Visual models, somatosensory models, cognitive models, self models, and all models in the brain are reductions or informational embeddings that simplify and compress. Subtle illusionism is necessarily true of all models in the brain and must be true of consciousness as well. The following hypotheses are consistent with this subtle illusionism.

## Hypothesis 3: The Illusionist Version of the Integrated Information Theory

In the integrated information theory (IIT), consciousness occurs when a system contains information that is in a state of integration (Tononi, 2008; Tononi et al., 2016). A mathematical metric for integration, phi, has been proposed. Whether any infinitesimal degree of integrated information can result in consciousness, or whether a threshold must be reached, is debated. Although the theory contains many facets, I will not discuss them here.

By the terminology outlined in this article, IIT is a magical mind theory. First, it assumes that a subjective, phenomenal experience, as we report it, exists. Second, it proposes a condition under which that conscious experience emerges; it proposes an Arrow A. Third, the theory lacks a specific proposal for Arrow B, or for how the conscious feeling, having emerged, has the physical ability to imprint itself on neuronal activity, such that it can become represented in Bob's semantic content and verbal output.

Here I will outline a simple addition to IIT that converts it from a magical mind theory to an illusionist theory (i-IIT). It is unlikely that the proponents of IIT would agree with i-IIT, because the illusionist version specifically removes the magicalism that makes IIT intuitively comfortable. However, i-IIT retains the central observation that the property we call conscious experience has similarities to the state of integration of information. It also has the advantage of solving several problems of IIT that have caused some scholars to call it nonscientific (IIT-Concerned et al., 2023).

Suppose the level of integration of information within the brain's neural networks is indeed a coherent, measurable property (Item 1). If that property is physically measurable, then it can in principle be detected by neurons and turned into a model (Item 2) that depicts the current state of integration. That model is, as always, schematic. It is a reduction or simplification. The model informs higher cognition (Item 3), which can inform the speech network (Item 4). In this hypothesis, Bob says that he has a subjective, intangible, phenomenal experience, because that is the schematic, reduced way in which Item 1 has been represented. In this modified version of IIT, one could not accurately say that integrated information is consciousness. Instead, a more accurate and careful description would be that integrated information is Item 1 in the chain of events that leads people to claim they have consciousness. In this hypothesis, consciousness resembles integrated information because consciousness is a distorted representation of integrated information.

One advantage of i-IIT is that it does not have a magical, intangible essence at the heart of it. The hard problem is dissolved. The subjective, phenomenal experience that Bob describes is a distorted construct. A schematic model represents integrated information in that distorted manner and therefore that is the property that Bob claims to have. Without the model, integrated information by itself would have no relationship to consciousness.

A second advantage of i-IIT is that it solves the problem of panpsychism (Pennartz, 2022). Since all things in the universe contain some degree of integrated information, therefore, in at least some interpretations of IIT, all things have consciousness. From a spiritual, religious, or wish-fulfillment perspective, panpsychism may be a desirable feature—and many scholars are especially drawn to this aspect of IIT. But for a scientific theory of consciousness, the claim that all things in the universe are conscious is unhelpful as well as untestable. It renders consciousness an essentially meaningless concept. In i-IIT, the problem of panpsychism disappears. Many objects in the universe contain integrated information, but without the associated neural embeddings and network processing, those objects have no relationship to consciousness.

For all its advantages, i-IIT does have potential limitations. First, it is not clear if the state of integration of information in the brain can be detected by neurons, such that it can be rendered into a model. Second, the adaptive use of the model is not clear. The brain builds models of items that are useful to monitor and predict. For example, the brain builds a model of the arm—the arm schema—because monitoring and predicting the state of the arm is useful in the control of movement. But what is the selective pressure for the brain to “know” about its own state of integrated information? Why would that model be constructed? It is possible that a continuously computed model of the state of integration of information in one's own brain would serve an as-yet undetermined, useful cognitive function. But the utility argument may be stronger for the hypotheses proposed next.

## Hypothesis 4: The Illusionist Version of the Global Workspace Theory

In the global workspace theory (GW), a select set of information in the brain reaches a state of signal enhancement, sometimes called ignition, in which the information is able to be broadcast widely to affect many systems around the brain (Baars, 1988; Dehaene, 2014; Mashour et al., 2020). This select set of information is said to be in the global workspace and by being in that state, has entered consciousness. In the global neuronal workspace theory (GNW), the workspace is centered specifically on the brain's parietal-frontal networks (Dehaene, 2014).

GW, at least in its simplest form, is a magical mind theory. Once the apple information reaches Bob's global workspace, it enters his consciousness. In this way, GW proposes Arrow A that gives rise to a mental experience. But where is Arrow B? How does Bob's brain acquire the semantic content, or make the verbal claim, that he is having a subjective experience? Another way to put the difficulty is that GW corresponds to the condition shown in Figure 1. Information about the apple is processed in visual cortex, enhanced by attention mechanisms to the extent that it reaches the parietofrontal networks, and from there it can impact downstream systems such as speech networks. This diagram shows how Bob processes visual information and can therefore say, “There is an apple and it is red.” However, GW does not encompass the condition shown in Figure 2. The information in the global workspace is all about the apple. Where did Bob's information about experienceness come from? How does he know to say, “And I have a subjective experience of the apple”?

These difficulties are immediately solved if we convert GW into an illusionist account. Suppose that in addition to having a global workspace, Bob's brain also constructs a model of its own global workspace. Since the global workspace has outputs that affect other parts of the brain, it is possible in principle for a system in the brain to gain information about and build a model of the global workspace. The model is, as always, schematic. It is a reduction or simplification. Bob says that he has a subjective, intangible, phenomenal experience, a theater of consciousness into which the apple or other objects can enter, and the theater of consciousness empowers him to choose and act and speak. Bob “knows” this is true, and says it, because that is the schematic, reduced way in which that model represents his global workspace. In this illusionist version of GW (i-GW), the global workspace is Item 1. The model, Item 2, is responsible for adding the more magical, phenomenological spin to the account that Bob ultimately gives.

A curious feature of i-GW is that Item 3, the higher cognitive network, is also the global workspace. Item 1 (the item being modeled) is the global workspace; Item 2 (the model) is a depiction of the global workspace; and Item 3 (the cognitive network that receives information from lower-level models) is, once again, the global workspace. In a somewhat mind-bending recursive twist, the global workspace contains simplified information about itself. It contains information about the apple and about how the global workspace contains the apple. The global workspace influences other systems such as speech output and it contains information about how it influences output systems. This interesting recursive structure explains how Bob can report on the apple (since the visual apple information has entered the global workspace) and yet also, separately, report on his state of experience of the apple (since the information about “experienceness” has also entered the global workspace). It is the key to understanding how Bob can say, “There is an apple,” as shown in Figure 1, and yet also be able to say, “I have a subjective experience of it,” as shown in Figure 2. Without that recursive property—if the global workspace did not contain a model of itself—we would be creatures that can talk about apples and other specific external content, but would never know about or talk about our own consciousness. With it, we become the philosopher creatures that we are, who can take notice, ponder, and argue about consciousness.

What is the adaptive advantage of building a model of the global workspace? Why would it have evolved in the first place? The answer may be that any controller performs better if it has a model of the thing it controls. In the end, the brain is a controller of behavior, and the global workspace is almost 100% determinative of behavior. Items that get into the global workspace can influence behavior, and items that do not rise to the level of the global workspace have at most a peripheral impact on behavior. Therefore, to guide one's own behavior, it would be useful to build a predictive model of one's own global workspace. It is, in effect, a model of me, of the most central, determinative parts of my brain's functioning, a model of what it means for me to fully know things in the moment and to make decisions and choose actions. The model, being schematic and incomplete, does not depict me as a complex biological machine. It instead depicts me as a magical essence inhabiting my body, capable of subjective, phenomenal experience and of a conscious will. The model tells me that I have consciousness because that is a useful, simplified way of keeping track of my global workspace.

One might ask, granted that the model is useful for the brain to construct, nonetheless, how does a person become conscious of the model? In an illusionist theory, the answer is that people do not become conscious of the model. Instead, the model contains information. The information enters higher cognitive networks and affects downstream systems, causing the person to semantically believe, and claim, whatever is depicted in that model—in this case, that a state of conscious experience is present.

## Hypothesis 5: The Attention Schema Theory

Each illusionist hypothesis described above is a guess about the identity of Item 1, the unknown item that is represented by a model (Item 2) in Bob's brain, leading to a semantic embedding in Bob's higher cognitive networks (Item 3), and from there to a speech output (Item 4) in which Bob claims that he has consciousness. So far, the proposed identities of Item 1 include an actual, magical mind (deemed unlikely here); nothing at all (the hard illusionist perspective, also deemed unlikely); the amount of integration of information in Bob's brain; and a global workspace. Can we find a systematic method for querying a model in the brain and identifying the thing that it models?

Take a well-understood case: the brain's model of the arm. The brain builds a body schema automatically and continuously (Head and Holmes, 1911; Graziano and Botvinick, 2002; Holmes and Spence, 2004). That model has been studied for more than a century, but how can one be sure that it actually represents the body, or that one particular part of it represents the right arm as opposed to the left leg or the head? The main reason is that when Bob closes his eyes and says, “My arm is up in the air right now,” it turns out that his arm is, usually, up in the air. The model covaries with the arm.

Consider the following simple hypothetical procedure. A scientist asks Bob to close his eyes and report what he can about his body. Bob says, “I have a central trunk and a set of appendages.” By asking Bob to introspect, the scientist is gaining information via an indirect route. The information inside a deeper model, the body schema, is passed through Bob's higher cognition and then his speech networks, to be received by the scientist. The content of the speech output may not be a perfect reflection of the deeper body schema—it may be an imperfect throughput—but it is one of the few easy ways for the scientist to listen in on Bob's body schema.

The scientist says, “Bob, tell me about one of those appendages. Pick one – I want to see if this internal model of yours matches anything externally measurable.”

Bob says, “I have an appendage attached to my right shoulder. It's about a meter long. It has a joint in the middle and little sticks at the end.”

The scientist excitedly measures Bob's right arm and says, “That description at least superficially resembles a real object. Tell me more.”

Bob says, “The appendage can move around. Now it's down at my side. Now it's up in the air.”

The scientist watches the movement of the arm and scribbles on his notepad, saying, “Yes, that internal model in your brain, as filtered through your cognition and verbal report, appears to covary with a real, measurable object – your right arm. Clearly, it's a model of your right arm.”

A second scientist, who is more skeptical, says, “But is it really a model of the arm? The arm has muscles, blood vessels, and bones. Bob, can you introspect in more detail and accurately tell me about those details of your arm?”

Bob says, “Introspectively, I don't know about those granular details. I can only tell you about the overall shape and position of this appendage.”

The skeptical scientist says, “Clearly the model lacks many of the details that the real arm has. And even worse, I can devise a situation in the lab such that the real arm is angled to the right, but Bob says it's to the left. Or, worse yet, if we cut Bob's arm off entirely, we know he's likely to have a phantom limb, i.e., the model persists even when the arm is gone. Therefore, that model doesn't always perfectly track the state of the arm. Are we sure it's a model of the arm? Maybe it's a model of something else. Is it a model of a leg? A model of somebody else's arm?”

A third scholar, who is more mystical-minded, chimes in. He says, “I think that Bob has an ethereal, invisible arm made of ghost essence. Sure, the ghost arm happens to move around roughly as his physical, objectively measurable arm moves. But the ghost arm is a separate thing. That's why it's still there after the real arm is amputated. Maybe it's a quantum vibration. Maybe it's an electric field, or a new ghost field that should be added to the standard model. The ghost arm is currently beyond the ability of science to measure, but Bob can nonetheless introspect and tell us about it – because, for reasons unknown, the ghost arm can physically impact Bob's cognitive and speech networks.”

I will risk the bold assertion that the mystical scholar is wrong. Yet the correct answer is not perfectly straightforward either. The skeptical scholar has a point—the real arm as measured objectively, and the arm that Bob describes on the basis of introspection, are not identical. How can we make sense out of the confusion? The answer is to adhere to a few principles. The brain's internal models are schematic and imperfect. Therefore, Bob's introspective account, informed by his arm schema, will not perfectly match the real arm. The match is still apparent—but it is just not perfect. The arm that Bob describes resembles the real arm in that both have a joint in the middle and a hand at the end. Moreover, the model accurately tracks the movement of the real arm a good 98% of the time. Therefore, it is a reasonable inference that when Bob closes his eyes and reports on the appendage sticking out of his right shoulder, that report derives from an internal model of Bob's actual right arm. It's not a model of a leg after all, because the properties it depicts do not resemble a leg and it does not covary with either of Bob's legs.

The logic is so obvious in the case of the arm that my explanation reads like an unnecessary belaboring of the point. Maybe the logic can be equally obvious in the case of consciousness.

As diagrammed in Figure 2, there is an unknown Item 1; it is represented by a model, Item 2; the model informs higher cognitive networks, Item 3; and higher cognition informs the language network, Item 4. When Bob verbally describes his conscious experience, he is providing information from the output end of that sequence. But how do we identify Item 1, at the start of the sequence? We’re looking for something with three diagnostic properties. First, Item 1 should have the physical ability to impact the activity patterns of neural networks. Otherwise, there would be no mechanism for detecting it and creating a model of it that can then affect cognition and speech. Second, Item 1 should resemble the consciousness that Bob describes having, at least in its superficial properties. We do not expect it to perfectly match conscious experience, because no model is a perfect description. In particular, we should expect the actual Item 1 to contain granular, physical details that are missing from Bob's account of consciousness. Despite those differences, the general, functional outlines of Item 1 should resemble the outlines of conscious experience as Bob describes it. Third, the real Item 1 should change state as Bob's account of his consciousness changes. It should covary with Bob's reported consciousness almost all of the time. If we can find something that satisfies those three constraints, then we could reasonably conclude that we have found Item 1, the real item that is the ultimate cause of Bob's claim that he has conscious experience.

I suggest there is a well-known item that matches those characteristics. Selective attention is a complex neural process, mainly in the cortex and thalamus, in which chunks of information that are encoded in neural networks compete with each other for greater signal strength (Desimone and Duncan, 1995; Kastner and Pinsk, 2004; Beck and Kastner, 2009; Moore and Zirnsak, 2017). Attention on the apple means the apple's representation in the brain is enhanced and can have a bigger downstream effect on the rest of the brain.

Attention appears to match the three diagnostic properties described above. First, because attention is a physical process that can have a measurable effect on neurons, it can be detected or transduced into an informational embedding. The brain would be able to construct a descriptive model of its own state of attention. Second, in their superficial properties, attention and consciousness resemble each other. The similarity is not perfect. Attention, after all, involves granular physical details such as neurons, synaptic inhibition, and specific pathways through the brain. Bob, describing his consciousness, might say that it is a what-it-feels-like essence, a vividness or immediate presence, that has no obvious physical moving parts—and that once something is in his consciousness, he can then make decisions about it. Bob's description of consciousness reads like a high-level, somewhat abstracted description of the mechanistic process of attention.

As for the third diagnostic property, we now have a hundred years of data showing that selective attention covaries with people's claims of conscious experience (James, 1890; Posner, 1994; Merikle and Joordens, 1997; De Brigard and Prinz, 2010; Prinz, 2012). If Bob says he was not conscious of the highway sign, he almost certainly had no attention on it, a phenomenon called inattentional blindness (Mack and Rock, 1988; Simons and Chabris, 1999; Drew, et al., 2013). If Bob says he was conscious of the highway sign, he almost certainly had at least some attention drawn to it, if only partial and peripheral attention. Attention and conscious experience covary so closely that it is difficult, though possible, to design a laboratory condition in which they are pried apart (McCormick, 1997; Kentridge et al., 1999, 2008; Norman et al., 2013; Webb et al., 2016b; Wilterson et al., 2020).

I suggest that the relationship between attention and consciousness has been staring science in the face for decades. But for the allure of mystical thinking and prior assumptions, it would be as straightforward as the relationship between the arm and the arm schema. Others have noted that attention and consciousness tend to covary (James, 1890; Posner, 1994; Merikle and Joordens, 1997; De Brigard and Prinz, 2010; Prinz, 2012). Here I offer this simple explanatory hypothesis. Attention is Item 1. It is a process of selectively enhancing some representations over others. Bob can pay attention to color and shape, to a sound, to a thought, to a memory, and so on. The brain not only uses the process of attention but also constructs a descriptive model of its own state of attention, which is functionally useful for the control of attention. That model, the attention schema, is Item 2.

Just like a visual model depicts the apple, and the body schema depicts the body, so the attention schema depicts attention in an automatic manner. Bob does not intellectually choose to think of himself that way. He can't help it. The attention model happens automatically and continuously. It is also not a detailed or accurate account of the mechanics of attention. There is no evolutionary pressure for the brain to know about neuronal competition, synaptic inhibition, the dorsal attention cortical pathway, or other mechanistic details. What this model depicts, instead, is an amorphous, mental essence, a mind vividly possessing something in the moment. That simple depiction works well enough for keeping track of attention. The model can then inform Item 3, the higher cognitive network. Higher cognition can influence Item 4, the speech networks. As a result, Bob claims to have conscious experience.

The attention schema is named in analogy to the body schema, and the analogy may involve more than a superficial similarity. Just as the brain needs a model of the arm to help control the physical appendage of the arm, which moves around and grasps objects, so it may need a model of attention to help control the virtual appendage of attention, which also moves around and grasps objects in its own way. In that sense, the attention schema is an extension of the body schema. Connections between the body schema, embodiment in general, and consciousness have been proposed before (Damasio, 1999; Blanke and Metzinger, 2009).

It has also been noted (Graziano et al., 2020) that AST is a version of a higher order thought theory (HOT). In HOT, the brain is conscious of X if the brain generates a higher order thought about X (Rosenthal, 2006; Gennaro, 2012; LeDoux and Brown, 2017). A higher order thought, or a metacognitive thought, is, one might say, the process of thinking about thinking. AST is a form of HOT because it postulates a model of attention. Attention is a cognitive process; constructing a representation of something is a cognitive process; and therefore to construct a model of attention is to apply one cognitive process to another. In AST, the visual model of the apple is a lower or first order representation, and the model of the act of attention on the apple is a second order or higher order representation. The model of attention is, however, not higher order in the sense of an explicit, or volitional thought. It is not that people think to themselves, “Let's suppose I’m conscious today.” Instead, the model of attention is just as automatic as the body schema, or as a visual model. When Bob looks at an apple and deeper systems in his brain supply his higher cognitive networks with a picture of reality in the moment, that picture includes a model of an apple and a model of attention. The picture of reality says, “There is an apple, and I have a conscious experience of it.”

One might call AST an illusionized version of HOT, or i-HOT. In AST, a conscious experience does not emerge from having a higher order thought. Instead, all claims that a person makes depend on information in the brain, and the model of attention is the specific bundle of information that serves as the basis for the claim, “I have a conscious experience.”

## Hypothesis 6: Deep Illusionism, or Many Keyhole Perspectives on a Deeper Theory

The most important suggestion in this article may be that many of the disparate hypotheses described here are not actually separate. Though they come from different points of origin, they converge on a deeper concept. The reason for that convergence is that a deep connection exists between selective attention, the global workspace, and the integration of information.

Attention and the global workspace are closely related. Attention involves the enhancement of representations in the brain, which then have a bigger effect on downstream systems. Attention can operate at many levels in the visual hierarchy, including the thalamus, primary visual cortex, and many higher layers of cortical processing (Desimone and Duncan, 1995; Kastner and Pinsk, 2004; Beck and Kastner, 2009; Moore and Zirnsak, 2017). Similarly, in GW, attentional enhancement boosts information to the highest hierarchical levels of processing in parietofrontal networks, where it can have a bigger effect on downstream systems. The global workspace and attention are inseparable phenomena. In that perspective, a model of the global workspace is really a type of model of attention, and as such, i-GW and AST are really just different versions of the same theory.

The integration of information is also closely related to attention. As known since Treisman's pioneering work on feature integration theory, information becomes integrated in the presence of attention (Treisman and Gelade, 1980). When you attend to a visual stimulus, the colors and shapes, patterns and textures are integrated into a single object. The parts click together. When no attention is directed to the object, the visual components come apart and are no longer processed in an integrated manner. The color, shape, texture, motion, and so on become computationally separated from each other.

One could validly argue that attention, the global workspace, and integrated information are different descriptions of the same underlying object—the deep, selective processing of information in the cerebral cortex. It is like the proverbial blind men feeling the elephant, where one feels the trunk, another feels the body, and a third feels the tail. Suppose you take a photo of the elephant. Do you now have a picture of a trunk? Of a body? Of a tail? They are all components of the original object, and you will find them all represented in the picture. The “elephant” in this case is the deep selective processing of information in the cerebral cortex. The “picture” is the schematic model that the brain constructs. It should not be a surprise if scholars, trying to interpret that picture, say, “Consciousness resembles attention; but it also resembles the global workspace; and yet, again, it resembles integrated information. It also seems to covary with all three of those.”

Given all of these considerations, I propose here a final, combined, illusionist hypothesis: Deep Illusionism. In this hypothesis, Item 1 is the deep selective processing of information in the brain. The brain also builds a simplified, schematic model of that deep processing (Item 2). The model allows the brain to monitor, predict, and better control its own deep processing. The model is a source of input to higher cognition (Item 3). Higher cognition informs the linguistic network (Item 4). At the end of that game of telephone, in which the original Item 1 has been coded and recoded, what comes out the other end is Bob's account of his own conscious experience.

If this is how Bob is constructed, if this is how we are all constructed, then it is understandable if a camp of scholars insists that consciousness resembles the global workspace. After all, based on introspective access to that model, everyone believes that consciousness is like a theater of the mind, the stage for all the things we think about and choose to act on. Consciousness and the global workspace share so many properties, it is intuitive to suggest that consciousness simply is the global workspace (Baars, 1988; Dehaene, 2014; Mashour et al., 2020). Another camp of scholars might say that consciousness resembles attention. After all, consciousness is when the mind seizes possession of something in an intensified manner. It shares so many of the properties of attention that maybe consciousness is attention (James, 1890; Posner, 1994; Merikle and Joordens, 1997; De Brigard and Prinz, 2010; Prinz, 2012). A third camp of scholars might say that consciousness resembles integrated information. Everyone knows that consciousness is both rich and at the same time unified; and an integrated set of very diverse information is by definition both rich and unified. Therefore, maybe consciousness is integrated information (Tononi, 2008; Tononi et al., 2016). Yet another camp might argue that based on introspection, everyone knows that consciousness is a feeling, not a physical thing, and therefore no scientific theory can ever bridge the gap from mechanism to an intangible, phenomenal experience—the hard problem (Chalmers, 1995). The reason why these different camps arrive at their many suggestions is that they are all, in their own ways, examining the same, schematic, internal model, and they are noting different, though related features of it.

The deep illusionist hypothesis is therefore not just a theory of consciousness. It is also a fairly effective theory of the broader landscape of theories. It helps explain why scholars are prone to arrive at the proposals they do. A swarm of theories, each different from the others, some of them trending more materialistic and some more magical, all emerge from partial, clouded glimpses of the brain's picture of the elephant. They emerge from Item 3 (higher cognition) accessing Item 2 (a deeper, automatic model), which is itself a distorted, detail-poor picture of Item 1 (the deep, selective processing of information).

It is tempting to ask where, in this framework, consciousness resides. Is it Item 1? Item 2? Is Item 3 necessary for consciousness? Does consciousness emerge from the interaction between these items? What are the necessary and the sufficient conditions for consciousness? However, the question itself is poorly formulated and misses the point of the illusionist framework. The framework proposes that we are agents that say we have conscious experience; we say it based on semantic embeddings in higher cognitive networks that encode the presence of conscious experience; the semantic embeddings are based on information from an automatic, deeper model; and the deeper model provides a distorted, detail-poor description of real physical processes in the brain. One cannot point to a part of that framework and say, “That's where the consciousness essence lives,” any more than one can point to a part of a car and say, “That's where the drive-around essence lives.” Illusionism explains the behavior of the system without postulating the existence of a consciousness stuff that emerges from or resides in any part of the system.

## Why a Mechanistic Theory of Consciousness Is Important

In my view, there are now only two broad classes of explanation for consciousness: magic and illusionism. Most of the field of study is mired in magic. The purpose of this article is to add at least one small push back toward rationality. It is difficult to convince others who are already invested in a way of thinking, but it may be possible to reach those from outside the field and show them that the study of consciousness has options for a clear-headed scientific approach.

That scientific approach may be important for our technological future. Everything discussed in this article could have applied to artificial as well as biological consciousness. In the illusionist framework, Bob could just as well be a deep learning neural network. If it is possible to build artificial consciousness, then it is going to happen, if it is not already here. We will have to decide what to do with a world in which consciousness is not just in us but also in our everyday technology. According to some speculations, artificial consciousness is an important step toward building prosocial AI that is more cooperative (Graziano, 2019). According to other speculations, consciousness will render AI more likely to harm us (Fast and Horvitz, 2017; Leffer, 2023; Pesen, 2023). In yet a third perspective, artificial consciousness will turn technology into an object of moral consideration, a so-called moral patient, greatly complicating how humans should behave toward it (Metzinger, 2013; Ryland, 2021; Akst, 2023).

This article is not intended to discuss the very complicated topic of artificial consciousness or its social or moral implications. My final point is simply that it is now a matter of urgency that we dispense with the magic, the mysticism, the pseudoscientific populism and the wishful thinking, and instead understand what consciousness actually is at the mechanistic level, including what benefits and risks it unlocks. Understanding consciousness is necessary for our immediate future. For the past century, the science of consciousness has focused on intellectual, philosophical and even religious goals, as researchers hoped to gain insight into the human condition. However, like the invention of stone tools, controlled fire, or writing, understanding consciousness at the level of engineering is likely to have an explosive effect in a practical sense that dwarfs the philosophical issues.

## Synthesis

Reviewing Editor: Christoph Michel, Universite de Geneve Decisions are customarily a result of the Reviewing Editor and the peer reviewers coming together and discussing their recommendations until a consensus is reached. When revisions are invited, a fact-based synthesis statement explaining their decision and outlining what is needed to prepare a revision will be listed below. The following reviewer(s) agreed to reveal their identity: John Bargh, Michael Pitts.

We receieved two very detailed and exhausted reviews that need to be shared with the author in their full length instead of making a global synthesis. We leave it up to the author to decide to which extend the thoughts of the reviewers will be integrated in a revised version.

Reviewer 1

There's been a proliferation of new theories and approaches to consciousness (defined as subjective experience) over the past 10 years or so and this manuscript is a much needed integration and synthesis of some of the main models. It gets at the heart of how these various models account for, mechanistically, the fact of conscious experience. Some strong suggestions include the 'magical' nature of the traditional theory and also global workspace theory which according to the author do not account for self-awareness (awareness of being aware) of the contents of the conscious field. The synthetic part of the argument is that each of the major theories focuses on one important aspect of conscious experience -- the integration of features into coherent objects in the Treisman tradition, the workplace notion where conscious contents can be operated on by many different modules, and the illusionist notion in which conscious experience is an internal model of the external world and not the world itself. These models are compared and shown to share important features as well as flaws, and the author argues that if each of them added an illusionist layer, they would all be improved as theories and progress would be made towards a more general and unified theory, with broader acceptance in the field. In short this is a valuable and potentially highly influential paper and I support its publication.

That being said, I had some ideas for improvement and also a few questions. First, I appreciated the historical review and believe more of that is needed right now (as LeDoux Michel and Brown 2020 have also provided and noted). But I was surprised that the Gestalt movement of 100 years ago was not mentioned, when certainly they were pioneers of the 'going beyond the information given' internal model of the world idea. Second, Bruner's history of the cognitive revolution and early cognitive science (in Acts of Meaning, and elsewhere) points out that the original cognitive psychology movement focused on how the mind/brain creates meaning itself, and says that in the 1980s cognitive science took a more information processing approach that assumed already existing units of information (eg., Marr's book on vision, PDP models). Everything became computational, machine learning etc. but that just bypassed the creation of meaning itself. The author's examples here often have the flavor of this meaning creation, which is refreshing, and there is an opportunity here to make this point more strongly, as it is an important aspect of the global workspace, and information integration, that is left out of most modern accounts. Meanings are not just given to us by computational processes but, especially in complex real world situations and environments, part of the work of consciousness itself is to actively, not just passively, create meaning. This runs against the description of the visual system as entirely automatic (there are no page numbers on this paper, but it is the paragraph comparing apples to bananas, right before the From Self Model to Speech section).

Along with Gestalt psychology, the other (very odd) omission was higher order models, such as by Rosenthal and also Ledoux and Brown. Clearly they could fit in the manuscript easily but why weren't they discussed? Also and quite relevant to the AI consciousness debate is Damasio's position that some form of embodiment is needed to attain subjective experience, because of the self-reflexive nature of interoception -- of awareness of one's own internal state. There are nice parallels and connections to Attention Schema theory here of course, because of the body-schema nature of Damasio's model, and maybe these could be discussed in the AI section near the end.

Under the Hypothesis 1 section, I resonated with the final couple of paragraphs. Hakwan Lau says similar things at the end of his recent book In Consciousness We Trust. And that concerns the appeal of overt spiritualism and decreased funding for professional scientific investigation of these theories and issues in favor of mainly private funding for the flashier new theories that are much less grounded in the long body of work in this area (for example, the split brain, blindsight and amnesiac patient work from 40-70 years ago). It is a pity that so-called science is actually so driven by the intuitions of power brokers such as editors and grant funding agencies, but it has been a widespread problem for some time.

But as I've brought up blindsight and split brain evidence, how does the AST and other theories handle the blindsight and split brain phenomenon? The LeDoux et al 2020 paper gives a nice historical review of this. It would be extremely useful to readers such as myself if the various theories discussed here were shown how they could or could not handle that evidence. This is information that the patient does not have conscious awareness of or access to, yet has many implicit effects nonetheless. How does AST and the other models here account for that fact?

And finally, in the final paragrph under Hypothesis 4, it is argued that a model of one's own global workspace is useful to keep track of my global workspace. But to me this seems to be pushing the question back a level and not answering it. Fine that we develop a model of our own attention, or global workspace, or body etc -- but why do we have to be aware of that model? Yes the model itself can be useful, let's assume that, but we are still left with the question of why the individual needs to be aware of that model. This needs to be made very clear, I felt I was left hanging by the argument just ending where it did in this paragraph.

To conclude, yes a version of this interesting and helpful paper should be published and I just thought it could be even better if it addressed some or all of the above concerns. Oh -- and for the most part I liked the examples, the body schema 'where is my arm' one was great -- but the one about photocopies is way outdated, and there must be a more modern version of the 'copy of copy of copy' loss of detail that could be used there.

Reviewer 2:

The main thrust of this paper is to argue that any viable theory of consciousness (from a physicalist / neuroscience perspective) requires some amount of illusionism. Although similar proposals have been made in the past, unique to this paper are (1) the crystal-clear logic used to support this argument, (2) a useful conceptualization of the range of possible illusionist perspectives, and (3) a set of intriguing ideas about how existing theories of consciousness could be improved by incorporating an illusionist component, whether in isolation for individual theories or via unification across theories. This is an important paper that is likely to influence both future theoretical and empirical work on consciousness. My comments are mainly aimed at helping to improve the paper further, to extend its reach, integrate it more thoroughly with existing theories, and to clarify points that are likely to trip some readers up along the way.

Note: despite the review instructions saying that this review should be double-blind, the author's name was included on the title page of the manuscript. To achieve the next-best thing (or as some would argue, an even better approach), I've signed my review to make it fully transparent.

1) In the first several pages, the author provides a nice overview of "illusionism". In the second paragraph of the intro, a broad definition is offered: "in illusionism, consciousness is not exactly what we think it is". This is exactly right. However, a few paragraphs later, the author uses an analogy of photographing a house and running the photo through many imprecise photocopies to drive home the point about representations and models being somewhat illusory or caricature-like compared to the real thing they are representing/modeling. I'm worried that this analogy, as written, might end up doing more harm than good to the author's central argument.

First, it makes it sound like consciousness is the house and the goal of consciousness science should be to study the house, rather than the imprecise model of the house. This might be true for some forms of (extreme) illusionism, but the ones the author seems to advocate for later on in the paper call for investigations of the models as well as the things they are modeling. We shouldn't just look for the real house, we should look for the blurry photocopies and the real house and the relationship between the two.

Second, toward the end of the paragraph on page 5, the author writes, "Illusionism implies that there is nothing present and the brain has made a mistake - in effect, the picture of the house is fake and no house exists." I don't think this is an accurate statement and risks confusing illusions (things are not exactly as they seem) with hallucinations and/or delusions (false percepts or beliefs about things that don't exist). Consider changing to something like, "Illusionism implies that the brain has made a mistake, and mistakes are generally to be avoided. Caricature more clearly implies a simplified distortion of something, and that the whole phenomenon is not just a brain mistake but serves a useful purpose."

2) Throughout the paper, I kept wondering how this all relates to various "higher order theories" of consciousness. In some sense, a model of something is a higher-order representation of it. How does the illusionism advocated for in this paper (in hypotheses 3, 4, 5, &6) relate to higher-order theories? Might the i-GW be like a higher-order thought theory, while the i-IIT is like a higher-order perception theory? If not, how is this proposal different than higher-order style theorizing? When trying to explain AST to my students and colleagues, I have often been asked, 'ok, well isn't this a type of higher-order theory then'? Adding something about higher-order theories, whether to connect with them or distinguish the proposed ideas from them, would strengthen the paper.

3) There are some interesting parallels between Figures 1 &2 and what many scientists who study conscious vs. unconscious processing do in their experiments. For example, in many studies, stimuli (like the apple) are presented at threshold levels and after each trial, subjects are asked to perform an "objective discrimination task" (e.g., 2AFC, is it an apple or a tomato, as in Figure 1) followed by a "subjective awareness task" (e.g., rate your experience of the stimulus on a 4-point scale like the PAS to report whether you had a subjective experience and if so, how clear was it, as in Figure 2). In many cases, these two measures line up, but they can come apart in interesting ways, such as when performance on the objective forced choice task is above chance while the subjective ratings are at zero (no experience). According to the present proposal, would these two tasks tap into two different things in the brain as in Figures 1 and 2, the latter being the model/caricature/illusion?

4) Hypothesis 1:

In Figure 3, is Arrow A analogous to Chalmers' "hard problem" (how and why do certain patterns of neural activity lead to conscious subjective feelings?) and is Arrow B analogous to Dennett's "hard question" (and then what happens?). If so, it might be worth explicitly linking these arrows to these two important problems/questions. I agree that the field has been fixated on Arrow A and I like the arguments in this section about the critical importance of Arrow B.

I'm worried that by the end of the second paragraph, some readers will immediately protest that the "feeling of seeing" is encoded in perceptual (and perhaps emotional) networks and then cognitive systems can access this information. Such a reader might ask: what is pseudosciencey about that? In other words, why can't items 1 &2 (in Figure 3) be the same thing and both in the brain? Or alternatively, why can't item 1 be "neural encoding of conscious feelings" (phenomenal consciousness) and items 2-3 be "neural encoding of detecting conscious feelings" (access consciousness). A reader like this might insist that the author is building a strawman argument here against dualist thinking, and they are a physicalist through-and-through so it doesn't apply to them. You can have p-consciousness without a-consciousness, they might say, and both are a result of different neural processes. I don't personally agree with this hypothetical reader, but I mention it here in case it's worth anticipating this kind of protest and perhaps providing a counter-point ahead of time.

5) General point:

At various points in the paper, especially towards the end of the Hypothesis 1 section, there seems to be a little too much emphasis on "understanding the item that is being modeled" rather than understanding the modeling process itself and the relationship between the model and the thing it is modeling. In other words, many consciousness researchers would argue that the goal is to explain why conscious experience seems the way it does (and what neural processes result in this seeming). If some form of illusionism is correct, we need to explain both what is really going on, and how/why it seems different from this reality from our first-person introspective point of view. If the author agrees, I wonder if some parts of the paper could be lightly revised to make this clear.

As a potentially analogy: It looks like the sun is moving across the sky and the earth is stationary. To explain how it seems, we can't just say, "what's really going on is that the earth is spinning" and stop there. We also have to add to this explanation components of size and speed and distance and perspective to link reality (earth spinning, sun stationary) to how it seems from our vantage point (earth stationary, sun moving). In the same way, to explain consciousness, we need to understand what's really going on in the brain and also how and why it seems like it does from our perspective (which can be drastically different). I think this is largely in line with what the author is aiming for with the proposal to add illusionism to any viable theory of consciousness, but several parts of the paper give the impression that the goal should be to understand item 1 (what is the "real thing" that we are mistaken about)?

6) Hypothesis #2:

This section was rather brief and could be improved and expanded to connect more explicitly with the hard illusionist proposals that are out there. For example, do Dennett's and Frankish's proposals fall into this category? If so, do they differ from each other, and how?

End of second paragraph: Consider changing, "What if there is no Item 1? What if Bob's consciousness is entirely an illusion" to "What if there is no Item 1? What if Bob's consciousness is more of a hallucination or delusion as opposed to an illusion?" And then at the end of this paragraph, "In that case, consciousness is not only a delusion, but also limited to humans".

7) Hypothesis #3:

This is excellent. Here are a few small ideas for further improving the impact of this section:

It might be helpful to anticipate some of the likely counter-arguments from the IIT-camp and address them from the outset. They may argue for example that the key to solving consciousness is to address Arrow A (the hard problem), and it's not the job of their theory to explain cognitive access or how we come to form beliefs, make claims, or report on our conscious experiences. They may also argue that the proposed "distorted representations of integrated information" are one of the main draw-backs of using behavior and reports to study what we are conscious of and how much we consciously experience. According to them (e.g., Haun et al., 2017, NoC) simplified (distorted/reduced) outputs of perception are what is measured through behavior/reports, but that underestimates how much we really experience perceptually.

In some sense, the starting axioms that Tononi and colleagues use to develop IIT must be derived from Arrow B. These "self evident" truths about consciousness that almost everyone (except some philosophers) would agree with have to be based on information that is in the brain that is accessed by higher-cognition and then communicated through language. Similarly, the "pure" consciousness that some claim to have experienced in certain altered states (like those Koch describes in his recent book) would be unknowable and unreportable if weren't for Arrow B. Similarly, Tononi recently wrote in his Neuron review paper, "when we dream, we can be vividly conscious even though we are not able to reflect, direct our thoughts and actions, and exert volition." This comes close to challenging the need for Arrow B (or at least the arrow between Item 2 and 3). But how would we ever know about and be able to report our dream experiences if it weren't for Arrow B?

In the last paragraph of this section, the author lists some potential weaknesses of i-IIT, namely, what would be useful (and adaptive) about having a model of one's own state of integrated information? I'm not sure this is really a weakness. I can think of at least 4 things off the top of my head that such a model could be useful for and therefore selected for in evolution: (1) a simplified model of the current state of integrated information could provide a very useful summary or sensory survey of our current situation which in turn could help guide decision-making and behavior in the immediate future, (2) such a model could form the content we use when we go "offline" in a kind of Kahneman-style "Type 2" thinking in order to flexibly and thoughtfully adjust our actions rather than responding reflexively/automatically to specific (less integrated) sensory information, (3) a model of integrated information could be used to assess our confidence in our perceptions, potentially leading to (4) so-called "reality monitoring". This is all to say that I'm not sure this paragraph makes a strong argument towards a weakness of i-IIT.

8) Hypothesis #4:

Again, this is an excellent proposal, with an especially convincing lead-up in the first few paragraphs. Just a few points to consider for improving clarity towards the end:

On page 23, "In this illusionist version of GW (i-GW), the global workspace is Item 1. The model, Item 2, ..." But then in the next paragraph, "In i-GW, the model not only depicts the global workspace, but...also sends its output to the global workspace.... Another way to put this recursion is that Item 3 is modelled by Item 2; Item 2 then informs Item 3" As written, the reader may be confused about the potential contradiction here. Item 2 is clearly the model, but is the workspace supposed to be Item 1 or Item 3? These two paragraphs appear to say both.

9) Overall point of clarity:

According to hypotheses 3, 4, 5, and 6, is the author proposing that consciousness is the model (Item 2) or that consciousness is the interaction between Items 1-2 (and potentially 3 as well)?

For example, at the end of the Hypothesis #6 section, the author writes, "A swarm of theories...emerge from Item 3 (higher cognition) accessing Item 2 (a deeper, automatic model), which is itself a distorted, detail-poor picture of Item 1." Again, is the proposal that consciousness is Item 2, or is it that we have come to have an illusion about consciousness based on the interactions of Items 1, 2, and 3? Relatedly, are Items 1 &2 sufficient for consciousness according to the present proposal? It would be helpful to clarify a bit here.

10) Page 24-25. Consider rewording, "But can we do better than try a series of arbitrary guesses about Item 1?" At least GWT and (some would argue) IIT are based on substantial amounts of empirical data, so they are far from being just 'arbitrary guesses'. Many would argue that AST falls into the same category, i.e., there is growing empirical support, but the relationship between an attention schema and consciousness is just as much of a 'guess' at this point as the relationship between the GW or II and consciousness.

11) Page 30. "I suggest that the relationship between attention and consciousness has been staring science in the face for decades." I love this suggestion and couldn't agree more! But keep in mind that AST is not entirely unique in this sense. GWT, for example, includes a very special role for attention, i.e., it's the key mechanism that determines what gets into the workspace. Other theories, such as Prinz's AIR also propose a critical role for attention. AST is certainly unique in the sense of the schema and its proposed relationship to awareness, but not in its proposed link between attention and consciousness.

Relatedly, on page 31, "Arguably, the global workspace is attentional enhancement as it applies to the highest hierarchical levels of processing, in parieto-frontal networks." This could be fleshed out in more detail, as it's unclear and potentially misleading. Does this mean a special type of attention is what determines entry into the GW, or is the author proposing that the broadcasting/ignition/information-sharing part of GWT is incorrect? If the latter, then is the idea that the content remains in perceptual networks while the job of the GW is to enhance these representations via attention? In other words, is the suggestion here taking the standard GW explanation at face value (perceptual information that is selected by attention is broadcast within a GW, thereby making it accessible to a wide range of cognitive systems) or is it proposing a new take on what the so-called GW actually is (e.g., it's an attentional network that operates on perceptual information represented elsewhere, but not broadcast within it)?

12) Hypothesis 6 is very interesting and potentially helps resolve a confusion I've had about AST. In several of the author's previous papers on AST, it was unclear whether the attention schema is just supposed to model attention or also the sensory/perceptual content that is attended. In some papers, like the response to critiques of the 2019 AST paper, where the author simplifies the problem to explaining how one comes to make the claim, "I am aware of X" with X being any content we can be conscious of, he seems to argue that AST only explains the "am aware of" part, while other models are needed to explain the "I" and the "X" parts. However, in other papers it seems like the attention schema is proposed as a model of both attention and the content that is selected/enhanced by attention. Is hypothesis 6 a type of unified theory that proposes a master-model of the "I" (GW), the "am aware of" (AST), and the "X" (IIT), all in one shot?

13) Hypothesis 6 also has some potentially useful supporting evidence from lesion studies that the author might consider mentioning:

If parts of the posterior hot zone (of IIT) are damaged, you get various different agnosias like prosopagnosia, akinetopsia, achromatopsia, object agnosia, etc. In some sense, the integration of certain kinds of sensory information is blocked by such lesions.

If parts of the attention networks are damaged, you get hemispatial neglect. Perhaps the attention schema is therefore disrupted.

If parts of the PFC component of the GW is damaged, you get disruptions to thought and action, with one of the most severe cases being akinetic mutism, or a cessation of voluntary goal-directed behavior.

These all seem to disrupt different aspects of consciousness, but via damage to perception, attention, or cognition components.

14) Although I found this paper to be quite interesting and thought-provoking, I keep getting frustrated about one thing: If these ideas are on the right track, then how do we proceed with consciousness research? We can't just measure and compare integrated information to global workspace to attentional selection, we have to study "models" or "schemas" of each of these. How exactly do we find these models in the brain and verify that they exist, what are the proposed neural mechanisms of the modeling process itself, and how do we determine whether one or two or three of these models best explains consciousness? I don't expect the author to be able to answer these questions in the current paper, but I wonder if a bit more can be said about next steps so that readers who are intrigued by these ideas at least have a clue about what we know, what we don't know, and what types of experiments would be needed at the next stage.

15) Small suggestion for the very end of the paper: Instead of basing the conclusion about why consciousness research is worth it narrowly on our technological future, why not also add something more broad, such as, "because of its centrality to our technological future and to our broader understanding of ourselves and our own existence in the natural world." I liked the final section about the urgency due to technological advancements, but if these illusionism x AST/GW/IIT ideas turn out to be correct, the payoff would likely be much broader.

Respectfully signed,

Michael Pitts

## Author Response

REPONSE TO EDITOR The editor states: We leave it up to the author to decide to which extend the thoughts of the reviewers will be integrated in a revised version.

My reply: Thank you for the flexibility. Most of these comments were helpful and I revised the manuscript accordingly. Some suggestions I did not implement, because I thought it would expand the scope of the paper too much.

RESPONSE TO REVIEWER 1 Thank you for a useful, deeply-thought set of comments. I incorporated most of your suggestions. A few of them, though good comments, I did not implement because I thought it would expand the scope of the paper too much.

Reviewer 1 states: this is a valuable and potentially highly influential paper and I support its publication.

My reply: I'm very glad you like the piece! Reviewer 1 states: I appreciated the historical review and believe more of that is needed right now. But I was surprised that the Gestalt movement of 100 years ago was not mentioned, ...

Bruner's history of the cognitive revolution and early cognitive science ... Marr's book on vision...

My reply: These are excellent suggestions. A historical perspective on consciousness should definitely include the Gestalt school. Marr is also important, especially in the modular approach to vision. These schools of thought are related to the discussion of models in the present paper.

However, much as I agree, I have backed off this particular change. I don't want this article to get bogged down in history. That wasn't in the original scope, and the piece is already about 50% over the recommended word limit. I'd like the piece to move rapidly away from introductory remarks and to get swiftly to the specific theories. In this passage in the introduction (especially paragraph 3), rather than give a history, I am really only making one point. Before computer technology, conceptions of consciousness focused on what was in consciousness - on the content of consciousness. Once computer tech came along, the content of consciousness became more easily explicable and even, in some ways, artificially duplicatable. Therefore, for scholars to maintain the mystery of human consciousness, a new concept was required: the hard problem, or the experiential essence. Thus the rise of Nagal and of Chalmers. My reading of the history is that the rise of computer technology was one of the main forces that led (through backlash) to the modern magical view of consciousness. I tried to rephrase, to clarify that I am making this one specific point.

Reviewer 1 states: Along with Gestalt psychology, the other (very odd) omission was higher order models, such as by Rosenthal and also Ledoux and Brown. Clearly they could fit in the manuscript easily but why weren't they discussed? 2 My reply: You are right. Higher order thought theories (HOTs) have a close relationship to AST.

I've revised to include two paragraphs on how AST relates to HOT (last two paragraphs of the section on AST).

Reviewer 1 states: Also and quite relevant to the AI consciousness debate is Damasio's position that some form of embodiment is needed to attain subjective experience, because of the self-reflexive nature of interoception -- of awareness of one's own internal state. There are nice parallels and connections to Attention Schema theory here of course, because of the body-schema nature of Damasio's model, and maybe these could be discussed in the AI section near the end.

My reply: Another good point. To address it, I added a short paragraph noting the connection between consciousness, the body schema, and embodiment, and citing Damasio, as well as Blanke and Metzinger (third-to-last paragraph of the AST section). However, to avoid a too-long paper being stretched even further, I avoided a detailed discussion.

Reviewer 1 states: Under the Hypothesis 1 section, I resonated with the final couple of paragraphs. Hakwan Lau says similar things at the end of his recent book In Consciousness We Trust. And that concerns the appeal of overt spiritualism and decreased funding for professional scientific investigation of these theories and issues in favor of mainly private funding for the flashier new theories that are much less grounded in the long body of work in this area (for example, the split brain, blindsight and amnesiac patient work from 40-70 years ago). It is a pity that so-called science is actually so driven by the intuitions of power brokers such as editors and grant funding agencies, but it has been a widespread problem for some time.

My reply: I'm glad we agree! It's an important point, I think.

Reviewer 1 states: But as I've brought up blindsight and split brain evidence, how does the AST and other theories handle the blindsight and split brain phenomenon? The LeDoux et al 2020 paper gives a nice historical review of this. It would be extremely useful to readers such as myself if the various theories discussed here were shown how they could or could not handle that evidence. This is information that the patient does not have conscious awareness of or access to, yet has many implicit effects nonetheless. How does AST and the other models here account for that fact? My reply: Here I'm going to disappoint the reviewer. I agree with the comment. However, I've written before in many places (e.g. my 2019 book) about blindsight, split brain, binocular rivalry, and especially hemispatial neglect. AST does an especially neat job of explaining these phenomena because they are all so closely related to attention and attentional control. However, to address these wide-ranging phenomena in a meaningful way that doesn't raise skepticism, but is actually convincing, requires a lot of space and a lot of explanation. I don't think this article should get into that level of experimental detail, or I'd have to go into many other lines of experimental evidence too. This article is only an introduction to potential theories and their links to illusionism. I'd prefer to leave these very important questions to another time and another place, or this article will be twice as long and the main points will be swallowed up.

3 Reviewer 1 states: And finally, in the final paragraph under Hypothesis 4... but why do we have to be aware of that model? Yes the model itself can be useful, let's assume that, but we are still left with the question of why the individual needs to be aware of that model. This needs to be made very clear, I felt I was left hanging by the argument just ending where it did in this paragraph.

My reply: The GW section in the paper was evidently not clear (reviewer 2 also commented on it), so I've rephrased parts of that section for better clarity, including adding a final paragraph to directly answer your question. You state, "we are still left with the question of why the individual needs to be aware of that model." In each illusionist theory, the proposal is always the same. People don't become aware of the model. Instead, the model contains information. The information depicts a state of awareness. The information enters higher cognitive networks and affects downstream systems, causing the person to think, and claim, whatever is depicted in that model - in this case, that a state of awareness is present. If the model instead contained non-sequitur information, such as, that a squirrel lives inside my skull, then higher cognition would be supplied with that invalid description of reality instead, and the person would think and claim that he had a squirrel in his head. One would not say, "we are still left with the question of why the individual needs a squirrel in his head." The answer is, he doesn't. He has information in his head. Hopefully the added paragraph helps to clarify.

Reviewer 1 states: but the one about photocopies is way outdated, and there must be a more modern version of the 'copy of copy of copy' loss of detail that could be used there.

My reply: You're probably right, but the photocopy example, though out of date, is still the simplest example I can think of, and audiences still know what it means.

RESPONSE TO REVIEWER 2 I thank reviewer 2 for a detailed, thorough, and useful review. Because this paper was supposed to have a word limit, and I have blown past it by 50% already, I can't take every one of the very good suggestions here, but I am grateful for them and will keep them in mind for future papers.

The reviewer states: despite the review instructions saying that this review should be double-blind, the author's name was included on the title page of the manuscript. To achieve the next-best thing (or as some would argue, an even better approach), I've signed my review to make it fully transparent.

My reply: Thank you for the transparency, and hello! In discussion with the editors, we decided that, given the content of this paper, it would not be possible to hide the fact that I wrote it, or to write it without using the first person in a few places. So there was no point in removing the author's name.

The reviewer states: 1)... the author uses an analogy of photographing a house and running the photo through many imprecise photocopies... I'm worried that this analogy, as written, might end up doing more harm than good... it makes it sound like consciousness is the house and the 4 goal of consciousness science should be to study the house, rather than the imprecise model of the house.

My reply: The reviewer has a point. I did not intend to imply that the only goal of consciousness studies is to study the house. Instead, the house and the model of the house go together. They are both important. I do maintain that a key question is: what is the item being represented? We must identify the house. But certainly another important question is: what is the nature of the imprecise representation? I revised the passage in question, and several other points throughout the manuscript, to clarify and to avoid the unintended implication.

The reviewer states: toward the end of the paragraph on page 5, the author writes, "Illusionism implies that there is nothing present and the brain has made a mistake - in effect, the picture of the house is fake and no house exists." I don't think this is an accurate statement and risks confusing illusions (things are not exactly as they seem) with hallucinations and/or delusions (false percepts or beliefs about things that don't exist).

My reply: In the revision, I rephrased to avoid confusion. You are right that illusionism doesn't always imply that nothing is present. However, it sometimes can. Your understanding of illusionism is more rational than some, and you're using a visual-science definition of illusion and hallucination. I have spoken to at least some illusionists who absolutely insist that the "subjective feeling" is 100% a construct, with no associated real process. Of course the visual information from the apple is processed in the brain, but according to some illusionists, the "experienceness" is a total fabrication - what you are calling a delusion. But more than that, outside of the illusionist camp, almost universally, people who hear about illusionism believe that it is advocating that consciousness is a total fabrication. I have a long passage in my 2019 book about why most people take the word "illusion" to mean that "nothing exists," even though that is not what it means in the technical sense of a visual illusion. It has to do with the way words are used metaphorically. Popular metaphors are culturally defined and widely used. When people say X is an "illusion," but they use the word metaphorically, what they mean is that X does not exist. Honesty in politics is an illusion. His kindness was an illusion. My hopes and dreams were illusions. This is how the word is colloquially used. As a result, when people hear the phrase, "Consciousness is an illusion," they assume the phrase means that there is no consciousness. There is nothing there. This is one of the reasons why I find the word so tricky. In any case, I revised that passage, rewording to aim for better clarity.

The reviewer states: 2) ... How does the illusionism advocated for in this paper (in hypotheses 3, 4, 5, &6) relate to higher-order theories? ...When trying to explain AST to my students and colleagues, I have often been asked, 'ok, well isn't this a type of higher-order theory then'? Adding something about higher-order theories, whether to connect with them or distinguish the proposed ideas from them, would strengthen the paper.

My reply: Yes, exactly! AST is a specific form of a higher-order thought (HOT) theory. I find there are many perspectives on HOT. Some are more magical (a conscious feeling emerges when a HOT is present). AST is an example of something more concrete (people claim to have a conscious feeling because that is the self-picture contained in a very specific kind of HOT).

Indeed, one could say that AST is really an illusionist version of HOT, or i-HOT. Given this 5 comment and the comments of Reviewer 1, I've revised the manuscript by adding a passage on why HOT is relevant (last two paragraphs of the AST section).

The reviewer states: 3) ... in many studies... subjects are asked to perform an "objective discrimination task" ...followed by a "subjective awareness task"... According to the present proposal, would these two tasks tap into two different things in the brain as in Figures 1 and 2, the latter being the model/caricature/illusion? My reply: Yes, that's the idea. At threshold, the components sometimes come apart. You can have attention on a stimulus, discrimination of the stimulus, and even a response to the stimulus, without having constructed the model of attention. Thus, the system can report what the stimulus is, while at the same time reporting that there is no experience. I've done some of these threshold experiments as well. In this paper I stayed away from the topic because it is huge, complex, and distracts from the main line of argument in this (already too long) paper.

The reviewer states: 4) ... In Figure 3, is Arrow A analogous to Chalmers' "hard problem" (how and why do certain patterns of neural activity lead to conscious subjective feelings?) and is Arrow B analogous to Dennett's "hard question" (and then what happens?). If so, it might be worth explicitly linking these arrows to these two important problems/questions. I agree that the field has been fixated on Arrow A and I like the arguments in this section about the critical importance of Arrow B.

My reply: I've added phrases and references to point out these connections, in the part of the text that discusses Figure 3.

The reviewer states: I'm worried that by the end of the second paragraph, some readers will immediately protest that the "feeling of seeing" is encoded in perceptual (and perhaps emotional) networks and then cognitive systems can access this information.

My reply: Noted, and I kept it in mind during revision. There are two ways to consider this proposed errant idea that readers might have. One way is the magical way, in which the feeling itself emerges from visual cortex and then magically affects output systems (by way of arrow B), such that we can say we have it. I find that to be the most common proposal, but it is also logically flawed, as noted in the "magical mind" section of the paper. The second way to consider your proposal for what readers might think is actually rational and workable, though inelegant. In that second way, the visual networks encode information about the apple, and ALSO encode information about a feeling of seeing. In that way, the visual networks are constructing two related models. One model depicts the visual features of the apple. The second model depicts second-order features of how the person is processing the apple. Why the visual system would construct a theory-of-mind model, in addition to a visual model, is not so clear, but not impossible. That version of the theory also implies that there is not just one model that gives rise to claims of experience, but many models, one in the visual system and one presumably in every other system for which we claim to have subjective experience. It is an inelegant, but not impossible, hypothesis. And it is, in its own way, compatible with illusionism, because it is about models and information, not about an actual, ethereal essence of experience. The possibility is quite deep and fascinating, and worth discussing somewhere. But when I write about AST, when 6 I chase down these important issues and attempt to answer common questions, I end up with a literal book. So I will severely limit how much I address these kinds of issues in the present paper.

The reviewer states: 5) ... there seems to be a little too much emphasis on "understanding the item that is being modeled" rather than understanding the modeling process itself and the relationship between the model and the thing it is modeling. ... If the author agrees, I wonder if some parts of the paper could be lightly revised to make this clear.

My reply: You are right, and I have kept my eye on that wording during the rewriting. Yet I still maintain that the first task is to figure out what item is being represented. Then it becomes possible to understand the representation and why it might be of cognitive benefit.

The reviewer states: As a potential analogy: It looks like the sun is moving across the sky and the earth is stationary...

My reply: Wonderful point. It's so good that Aaron Schurger and I wrote a piece not too long ago that contained that exact analogy! The reviewer states: 6) Hypothesis #2. This section was rather brief and could be improved and expanded to connect more explicitly with the hard illusionist proposals that are out there. For example, do Dennett's and Frankish's proposals fall into this category? If so, do they differ from each other, and how? My reply: I understand the comment. In this case, however, I will choose to leave the section short and superficial. One reason is that I have no desire to explicitly attack illusionists.

Politically, I'd rather be vague here. Even Dennett and Frankish suggest a range of views and do not adhere to an extreme view. Moreover, this section really does not need to be meaty, because it is only a starting point needed to clarify the concept in the most extreme form, so that more subtle forms are easier to understand.

The reviewer states: End of second paragraph: Consider changing, "What if there is no Item 1? What if Bob's consciousness is entirely an illusion" to "What if there is no Item 1? What if Bob's consciousness is more of a hallucination or delusion as opposed to an illusion?" And then at the end of this paragraph, "In that case, consciousness is not only a delusion, but also limited to humans".

My reply: I understand your idea, and edited the wording to be more careful about the term illusion. I no longer say such things as "entirely an illusion" to mean "a model without any item that is being modeled." But as discussed above, I think when people use the term "illusion" metaphorically, they tend to use it as basically synonymous with "hallucination" or "delusion." It's one of the reasons why, in the past, I've been reluctant to use these words. In this article, I'm finally giving in and accepting the word illusion! But I am not sure that I'm ready to use these other terms without adding a great deal of definition and explanation. One of the problems with all of these terms is the common lay-person response: "If consciousness is a delusion (or hallucination), then what is the mind that is having the delusion? Does one not need a 7 consciousness first, to have a delusion? Is this not circular?" Of course it isn't circular, but it requires careful definition to make sure that nobody is confused or that nobody uses the confusion as a cheap line of attack.

The reviewer states: 7) ... Here are a few small ideas for further improving the impact of this section...

My reply: The reviewer provides a useful discussion of the IIT section of the paper (hypothesis 3). I have kept these comments in mind during revision. However, I'd rather not make each section a detailed discussion that raises and then defends against possible attacks. That would make for a very long paper, in which each section is an extensive essay on a different prior theory. Here, instead, I briefly mention a prior theory, then sketch the illusionist version of it, then move on. My hope is that readers will see something useful in those illusionized versions. I do not disagree with the reviewer here. A full, long, theory paper, dedicated entirely to IIT and its illusionized version would make a good contribution. That is a fantastic idea. The present article, alas, must go rapidly through many illusionist options derived from many prior theories, or risk turning into another book.

The reviewer states: In the last paragraph of this section, the author lists some potential weaknesses of i-IIT, namely, what would be useful (and adaptive) about having a model of one's own state of integrated information? I'm not sure this is really a weakness.

My reply: I revised the wording to make clear that this is a proposed challenge to the theory, but it is always possible for a solution to emerge. Mainly, in that passage, I am trying to point out one of the most important criteria for evaluating a theory of consciousness - is there an adaptive benefit? Why would it have evolved in the first place? The reviewer states: 8) Hypothesis #4: On page 23, "In this illusionist version of GW (i-GW), the global workspace is Item 1. The model, Item 2, ..." But then in the next paragraph, "In i-GW, the model not only depicts the global workspace, but...also sends its output to the global workspace.... Another way to put this recursion is that Item 3 is modelled by Item 2; Item 2 then informs Item 3" As written, the reader may be confused about the potential contradiction here.

Item 2 is clearly the model, but is the workspace supposed to be Item 1 or Item 3? These two paragraphs appear to say both.

My reply: I tried to revise for better clarity. The global workspace acts as item 1 (the thing being modelled). The global workspace also acts as item 3 (the higher cognitive representation). So it serves double duty. Which makes the dynamics kind of interesting and complex.

The reviewer states: 9) Overall point of clarity: According to hypotheses 3, 4, 5, and 6, is the author proposing that consciousness is the model (Item 2) or that consciousness is the interaction between Items 1-2 (and potentially 3 as well)? My reply: I added a paragraph to the end of the Hypothesis 6 section, and a few other sentences here and there, to help answer this question. I am carefully avoiding staking a claim. I see it as a philosophical tar pit and a moot question. Suppose I look at a chair and tell you, "I see a chair!" 8 You could get philosophical and say, "Actually, the chair is a construct in your mind. The physical item out there is technically something else and not a chair." Another commenter could say, "No, it's the opposite. The chair is the real thing out there. The representation in the brain is distorted and is not really a chair." I would say, let's stop the semantics and describe what is literally happening. "I see a chair" is an utterance that happens when there is a certain kind of real item, my brain represents it, the representation affects my higher cognition, and my higher cognition affects my speech. Exactly which part of the process should we label as the real chair? It doesn't matter. Whatever you like. Just so, the entire process I describe here results in an agent that thinks and says it has consciousness. What part of the multi-step process should we call the real consciousness? That's unimportant, and perhaps misses the point. I have tried, in the rewrite, to add a paragraph here and there to help clarify, in case it was not already clear.

The reviewer states: Relatedly, are Items 1 &2 sufficient for consciousness according to the present proposal? It would be helpful to clarify a bit here.

My reply: I added a paragraph to the end of the Hypothesis 6 section to help answer this question Philosophically, I'd say the very question is mistaken. The question itself is rooted in a traditional magical perspective. "Sufficient for consciousness" implies that consciousness is a thing that is present or not, depending on whether the right ingredients are here. In this paper I am explaining the conditions under which a real thing (perhaps attention?) is represented, then informs higher cognition, then results in claims about having consciousness. If a brain can only have items 1 and 2, then it has only half of that process. If you want to refer to that as consciousness, you may, although it would then not be the complete human version. If you ask, "Yeah, but would it have or lack an inner feeling?" the answer is, it would lack an inner feeling, just as we do. But unlike us, it would also lack the higher cognitive ability to think it has inner feeling or say it has inner feeling. It's a bit like asking, at what point in the construction process can you refer to a car as a car? With just the chassis? The whole body? The complete engine? The ability to actually be driven? My answer is, I don't know when the label of car should apply, and because it's an issue of terminology, it isn't scientifically interesting. Maybe it becomes interesting only if one thinks there is a magic consciousness essence that is present in some systems and absent in others. Then one can ask, which ingredients are necessary to make the magic essence emerge? See the new final paragraph of the Hypothesis 6 section.

The reviewer states: 10) Page 24-25. Consider rewording, "But can we do better than try a series of arbitrary guesses about Item 1?" At least GWT and (some would argue) IIT are based on substantial amounts of empirical data, so they are far from being just 'arbitrary guesses'. Many would argue that AST falls into the same category, i.e., there is growing empirical support, but the relationship between an attention schema and consciousness is just as much of a 'guess' at this point as the relationship between the GW or II and consciousness.

My reply: I rephrased that whole passage to help clarify. The point here, at the beginning of the AST section, is to outline a systematic method by which one can start with a representation and then work backward and figure out what it is a representation of.

The reviewer states: 11) Page 30. "I suggest that the relationship between attention and consciousness has been staring science in the face for decades." I love this suggestion and 9 couldn't agree more! But keep in mind that AST is not entirely unique in this sense. GWT, for example, includes a very special role for attention, i.e., it's the key mechanism that determines what gets into the workspace. Other theories, such as Prinz's AIR also propose a critical role for attention. AST is certainly unique in the sense of the schema and its proposed relationship to awareness, but not in its proposed link between attention and consciousness.

My reply: I agree. I make sure to cite Prinz and also to note the similarity between GWT and AST. The two are compatible and especially easy to integrate together, as pointed out in the Hypothesis 6 section of the paper. Note especially the sentence that consciousness "shares so many of the properties of attention that maybe consciousness is attention (James, 1980; Posner, 1994; Merikle and Joordans, 1997; De Brigard and Prinz, 2010; Prinz, 2012)." The reviewer states: Relatedly, on page 31, "Arguably, the global workspace is attentional enhancement as it applies to the highest hierarchical levels of processing, in parieto-frontal networks." This could be fleshed out in more detail, as it's unclear and potentially misleading.

Does this mean a special type of attention is what determines entry into the GW, or is the author proposing that the broadcasting/ignition/information-sharing part of GWT is incorrect? ...

My reply: I am merely suggesting that the standard GWT could also be described using the language of attention. I edited the wording to try to clarify.

The reviewer states: 12) Hypothesis 6 is very interesting and potentially helps resolve a confusion I've had about AST. In several of the author's previous papers on AST, it was unclear whether the attention schema is just supposed to model attention or also the sensory/perceptual content that is attended. ... Is hypothesis 6 a type of unified theory that proposes a master-model of the "I" (GW), the "am aware of" (AST), and the "X" (IIT), all in one shot? My reply: I think I have an even longer passage in my 2013 book on that topic. I usually refer to the attention schema as a model of attention itself, not the object being attended or the agent doing the attending. But there is, of course, a larger master-model, an integration of component models. The brain constructs a larger, integrated model, sometimes called a world model, only one component of which is the attention schema. That's how I see the terminology. But without any change to the actual underlying concepts, you could, if you like, redefine the term "attention schema" and make it refer to the larger model. However, in that case, you no longer have a label for the smaller component of the master model that represents attention itself. In any case, this question concerns terminology and not underlying concept.

The reviewer states: 13) Hypothesis 6 also has some potentially useful supporting evidence from lesion studies that the author might consider mentioning: If parts of the posterior hot zone (of IIT) are damaged, you get various different agnosias like prosopagnosia, akinetopsia, achromatopsia, object agnosia, etc. In some sense, the integration of certain kinds of sensory information is blocked by such lesions. If parts of the attention networks are damaged, you get hemispatial neglect. Perhaps the attention schema is therefore disrupted. If parts of the PFC component of the GW is damaged, you get disruptions to thought and action, with one of the most severe cases being akinetic mutism, or a cessation of voluntary goal-directed behavior.

10 These all seem to disrupt different aspects of consciousness, but via damage to perception, attention, or cognition components.

My reply: These are good points. I've certainly found hemispatial neglect very relevant in some of my prior work. I am less clear on whether agnosias represent a loss of integration, or a loss of specific high-level sensory processing. So the issues are a little complex, with some caveats, and the lesion data probably needs some nuance and discussion. For that reason, just to limit the scope of the paper and avoid making it longer than it already is, I will not delve into the lesion data. The paper remains an outline of some illusionist-type theories, without a deep-dive into the evidence for each one of them.

The reviewer states: 14) Although I found this paper to be quite interesting and thought-provoking, I keep getting frustrated about one thing: If these ideas are on the right track, then how do we proceed with consciousness research? We can't just measure and compare integrated information to global workspace to attentional selection, we have to study "models" or "schemas" of each of these. How exactly do we find these models in the brain and verify that they exist, what are the proposed neural mechanisms of the modeling process itself, and how do we determine whether one or two or three of these models best explains consciousness? I don't expect the author to be able to answer these questions in the current paper, but I wonder if a bit more can be said about next steps so that readers who are intrigued by these ideas at least have a clue about what we know, what we don't know, and what types of experiments would be needed at the next stage.

My reply: I know what you mean. I guess studying cognitive models has a long tradition, and my own experiments are attempts to get at it from many different angles. Since this is a theory paper, not so much a discussion of data, I don't delve into our experimental evidence here. My personal feeling, however, is that theories of consciousness will ultimately stand or fall on AI research. In artificial systems, some of these models and processes are more manipulable and measurable.

The reviewer states: 15) Small suggestion for the very end of the paper: Instead of basing the conclusion about why consciousness research is worth it narrowly on our technological future, why not also add something more broad, such as, "because of its centrality to our technological future and to our broader understanding of ourselves and our own existence in the natural world." I liked the final section about the urgency due to technological advancements, but if these illusionism x AST/GW/IIT ideas turn out to be correct, the payoff would likely be much broader.

My reply: I edited the wording at the end to help address this point. However, honestly, I think that the philosophical knowledge we gain is mostly unimportant up against the technological change. In my view, conscious AI is potentially the most transformative change to life on earth since it began, 4 billion years ago. What we humans philosophically think about consciousness is not so big in comparison.
